# Effect of abutment design on fracture resistance of resin-matrix ceramic crowns for dental implant restoration: an in vitro study

**DOI:** 10.1186/s12903-023-03100-0

**Published:** 2023-06-21

**Authors:** Jie Lin, Pingping Cai, Yingying Zhuo, Ling Lin, Zhiqiang Zheng

**Affiliations:** 1 Fujian Key Laboratory of Oral Diseases, School and Hospital of Stomatology, Fujian Medical University, 246 Yangqiao Zhong Road, 350002 Fuzhou, Fujian PR China; 2 Department of Crown and Bridge, School of Life Dentistry at Tokyo, The Nippon Dental University, 1-9-20 Fujimi, Chiyoda-ku, 102-8159 Tokyo, Japan; 3 School of Life Dentistry at Tokyo, The Nippon Dental University, 1-9-20 Fujimi, Chiyoda-ku, 102-8159 Tokyo, Japan

**Keywords:** Resin-matrix ceramics, Fracture resistant, Dental implant restoration, Crown thickness, Abutment

## Abstract

**Background:**

The purpose of this study is to investigate the performance and fracture resistance of different resin-matrix ceramic materials for use in implant-supported single crowns with respect to the abutment design (crown thickness: 1 mm, 2 and 3 mm).

**Methods:**

Forty-eight abutments and crowns were fabricated on implants in the right lower first molar. Two resin-matrix ceramic materials for dental crowns were selected for study: (1) a glass-ceramic in a resin interpenetrating matrix (Vita Enamic, Vita, Germany) and (2) a resin-based composite with nanoparticle ceramic filler (Lava Ultimate, 3 M ESPE, USA). Three types of abutments were designed: 1 mm thick crown + custom titanium abutment, 2 mm thick crown + custom titanium abutment and 3 mm thick crown + prefabricated titanium abutment. The experiment was divided into 6 groups (n = 8) according to the crown materials and abutment designs. After 10,000 thermocycles, fracture resistance was measured using a universal testing machine. The statistical significance of differences between various groups were analysed with ANOVA followed by a *post hoc* Tukey’s honestly significant difference test. The surfaces of the fractured specimens were examined with scanning electron microscopy (SEM).

**Results:**

Two-way ANOVA revealed that the abutment design (F = 28.44, P = 1.52 × 10^− 8^<0.001) and the crown materials (F = 4.37, P = 0.043 < 0.05) had a significant effect on the fracture resistance of implant crown restoration. The Lava Ultimate-2 mm group showed the highest fracture resistance of 2222.74 ± 320.36 N, and the Vita Enamic-3 mm group showed the lowest fracture resistance of 1204.96 ± 130.50 N. Most of the 1 and 2 mm groups had partial crown fractures that could be repaired directly with resin, while the 3 mm group had longitudinal fracture of the crown, and the crowns were detached from the abutments.

**Conclusion:**

Based on the in vitro data of this study, the fracture resistance of the 2 mm thick resin-matrix ceramic crown design was higher than that of the 1 and 3 mm groups. The 2 mm thick resin-matrix ceramic crown and personalized abutment are an option to replace zirconia for implant crown restoration.

## Background

In recent years, zirconia and porcelain-fused-to-metal (PFM) have been widely used in dental implant restoration. There is no periodontal ligament in dental implants; if a high-strength material such as zirconia is used for crown restoration at both the upper and lower sides, it will bring greater concentrated stress and long-term fatigue impact to the implant, which may affect the stability of the implant [1]. According to the reports of Sahin et al. [2] and Menini et al. [3], crown restoration with resin material can compensate for the high elastic modulus of the implant material, and thus it can reduce the impact of the implant on the surrounding bone. Natural teeth undergo physiological wear, while ceramic materials such as zirconia have high hardness and less wear; thus, long-term use and uneven wear between natural teeth may cause occlusal interference [4]. In addition, it was reported that the chipping of veneering porcelain in 5 years can reach 47% [5, 6].

Resin-matrix ceramics [7], as a new dental restoration material, have good physical, chemical and aesthetic properties. They can inhibit brittleness and improve machinability when in use, and they are relatively convenient for processing and subsequent treatment [8, 9]. Resin cement has a similar composition to resin-matrix ceramics. The research results of Peumans et al. [10] and Stawarczyk et al. [11] showed that sand blasting, hydrofluoric acid and silane coupling agent treatment could improve the bonding strength of resin-based ceramics. Compared with PFM or all ceramic crown restorations, resin-matrix ceramics have many advantages [12], such as strength similar to that of natural teeth and good biocompatibility, and they do not affect magnetic resonance imaging. The experimental results of Lawson et al. [7] showed that the physical properties and wear degree of resin-based ceramics are similar to those of natural teeth [8]. Therefore, when ceramic materials are used in the upper edentulous jaw, resin materials are often used in the lower edentulous jaw to avoid so-called “hard hitting hard” phenomenon.

There are strict designs for the preparation of natural teeth, and there are different standards and regulations for different materials. The abutment designs [13] of the axial surface, occlusal surface and incisal edge have been repeatedly studied and demonstrated, and the results provide strong guidance and support for the design of restorations by clinicians. However, many research of implant abutment design is focused on peri-implant soft tissues [14], there have been relatively few studies of the design of crown and abutment. In many cases, a custom abutment is completely designed by technicians. When using preformed abutments, there are no differences between materials and situations. Most implant abutments are made of pure titanium and zirconia with elastic moduli of 110 GPa and 210 GPa [15], respectively, which are much larger than those of natural teeth [16]. There is a certain difference between crown restoration supported by implant abutments and natural tooth restoration, and the preparation rules of natural teeth cannot be directly applied to implant abutment design.

The relative strength of resin-matrix ceramics is less than that of other crown restoration materials [8, 9, 12], and thus it is unclear whether they can withstand the occlusal force for a long time when applied to posterior dental implant crown restorations. The purpose of this study was to investigate the performance and fracture resistance of different resin-matrix ceramic materials as implant-supported single crowns with respect to the abutment design (crown thickness) and analyse the fracture mode.

## Methods

This experiment simulated the implant prosthetic design of the lower right first molar. Two resin-matrix ceramic materials (Table 1) for dental implant crowns were selected for study, each procured in the form of commercially available computer-aided design/computer-aided manufacturing (CAD/CAM) blocks: (1) a glass-ceramic in a resin interpenetrating matrix (Vita Enamic, Vita, Germany) and (2) a resin-based composite with nanoparticle ceramic filler (Lava Ultimate, 3 M ESPE, USA).

**Table 1:** Resin-matrix ceramic material composition and indication according to manufacturer’s data

Product name	Manufacturer	Shade (Lot no.)	Composition (manufacturers’ information)
Lava Ultimate,	3 M ESPE, USA	A3, (N366024)	80 wt% (65 vol%) nanoceramic particles (zirconia filler (4–11 nm), silica filler (20 nm), aggregated zirconia/silica cluster filler) 20 wt% (35 vol%) highly cross linked (methacrylate-based) polymer matrix Silane
VITA ENAMIC	VITA Zahnfabrik, Germany	A3, (100,003)	86 wt% feldspathic-based ceramic network 14 wt% acrylate polymer network (infiltrated into feldspathic-based ceramic network)

Twenty-four Vita Enamic and 24 Lava Ultimate CAD/CAM ceramic blocks were randomly divided into the following 6 groups (n = 8).

Vita Enamic-1 mm: 1 mm thickness Vita Enamic crown + custom titanium abutment.

Vita Enamic-2 mm: 2 mm thickness Vita Enamic crown + custom titanium abutment.

Vita Enamic-3 mm: Vita Enamic crown + prefabricated titanium abutment.

Lava Ultimate-1 mm: 1 mm thickness Lava Ultimate crown + custom titanium abutment.

Lava Ultimate-2 mm: 2 mm thickness Lava Ultimate crown + custom titanium abutment.

Lava Ultimate-3 mm: Lava Ultimate crown + prefabricated titanium abutment.

### Abutment and crown manufacturing

The crown data of the standard form of the right lower first molar were reduced through a dental CAD software package (Cerec premium SW 4.5, Dentsply Sirona, Germany), and the custom abutment design with reserved crown spaces of 1 and 2 mm was conducted. The custom titanium abutments (Preface abutment, Medentika, Germany) were made by cutting with a lathe (RKO8, Germany). The transmucosal height was 2 mm, and the cement space was set to 50 μm. The prefabricated titanium abutment used an adhesive retention abutment (022.4326, Straumann, Switzerland) with a diameter of 5.0 mm. The transmucosal height was 2 mm, which was consistent with the custom abutment. The axial space of the reserved crown restoration was approximately 3 mm. The abutment was connected with the substitute through the central screw, and the applied torque was 35 Ncm. All abutment and crown restorations were made by the same technician.

Eight blocks of each group were machined into anatomically correct right mandibular first molar crowns by standard dental dies of Cerec premium SW 4.5 CAD software into the CEREC System (Dentsply Sirona, Germany). The crown thicknesses in the custom titanium abutment groups were 1 and 2 mm. The occlusal crown thickness and proximal wall thickness for the prefabricated abutment group were 1 and 3 mm, respectively. Allowance was made for a cementation thickness of 50 μm in all cases. The dies were engineered to provide common external crown dimensions for all materials; all crowns had the same shape and size, while abutments had different sizes.

### Pretreatment of crown and abutment

The crowns were sand blasted with alumina particles with a diameter of 50 μm [17, 18]. The pressure was 0.1 MPa, the distance was 1 cm for 15 s. The titanium abutments were also sand blasted with alumina particles with diameter of 110 μm [19]. The pressure was 2.5 MPa, and the distance was 1 cm for 60 s. The crowns and abutments were cleaned with cotton pellets and 100% alcohol for 5 s and then air dried for 5 s.

Universal adhesive (Single bond universal, 3 M ESPE, USA) was applied with a disposable small brush on the bonding surface of each crown and abutment according to the instructions provided. The adhesive was cured for 20 s, dried with dry air for 10 s [20], and illuminated on each side by a curing lamp (Elipar S10, 3 M ESPE, USA) for 5 s.

### Placement of restorations

Resin cement (RelyX Ultimate, 3 M ESPE, USA) was mixed according to the manufacturer’s instructions and evenly applied to the inner surfaces of the dental crowns. After surface treatment, the crown on the abutment were luted together with the RelyX Ultimate, with a constant load of 9.8 N to the central fossa for 10 min [21]. The load direction was consistent with the implant long axis. According to the operating instructions of the RelyX Ultimate provided by the manufacturer, excess resin adhesive was removed after the adhesive was cured, and then an oxygen inhibitor was coated on the edge of the crown. Then, the four sides and the top were irradiated with a curing lamp for 10 s each.

### Thermocycling, fracture resistance and fracture modes

To simulate the oral environment, specimens were stored in an incubator from 24 to 48 h at 37 °C at 90% humidity until thermocycling (TC-501f, Weill, China) commenced. The restored teeth were subjected to 10,000 thermal cycles in alternate water baths at 5 and 55 °C with a 20 s dwell time for each bath [20].

**Fig. 1:**
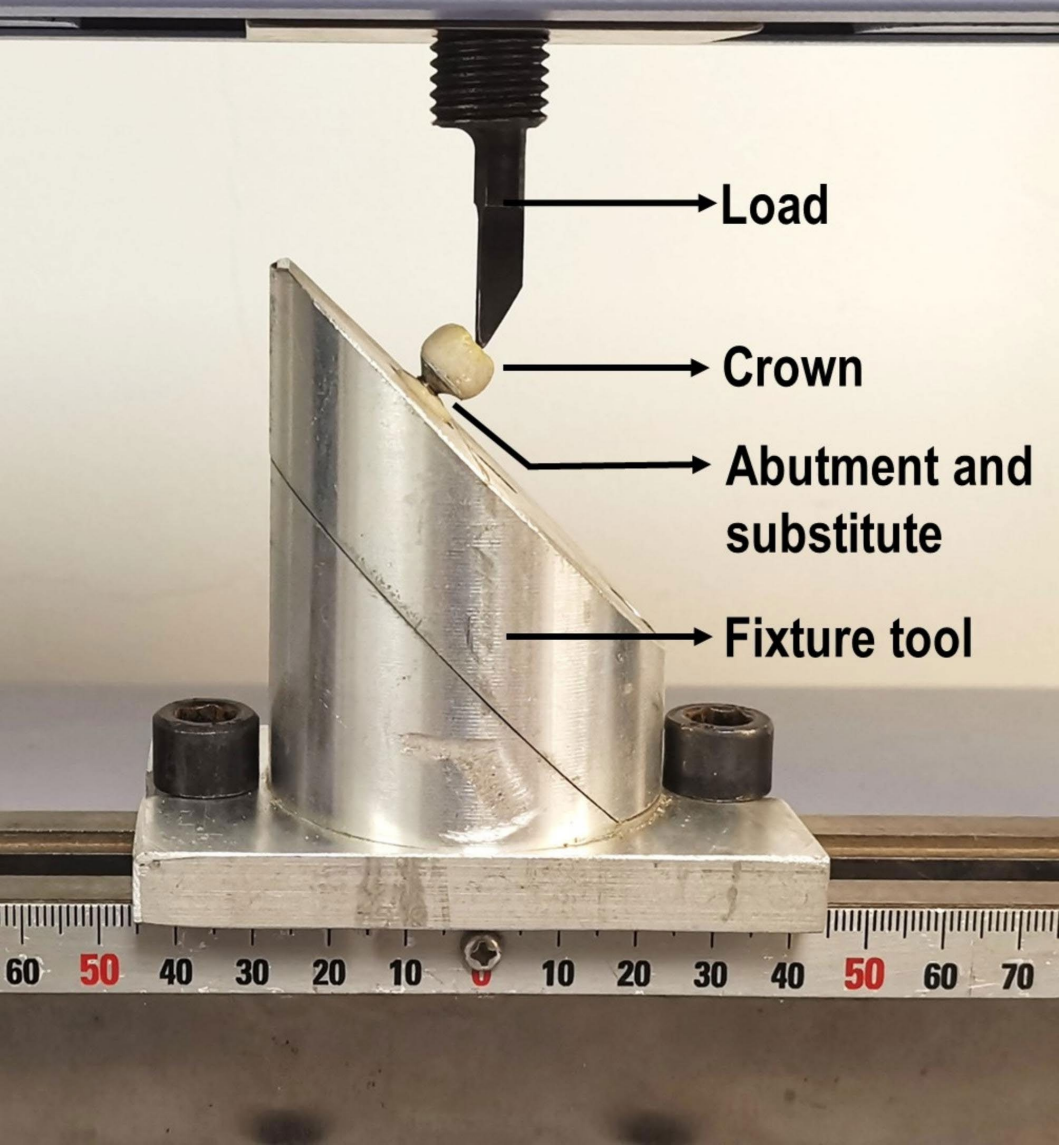
Universal testing machine loading mode, position and direction

After thermocycling, fracture resistance was measured using a universal testing machine (AGS-X, Shimadzu, Japan). Each specimen was inserted into a custom-made holding device (Fig. 1), and the fracture resistance of each specimen was measured. The force was applied to the buccal cusp through a spherical cast NiCr alloy loading head at a 45° angle [20] with the long axis of the implant, and the loading displacement speed was 1.0 mm/min [22]. The standardized crown configuration allowed each specimen to be installed in the same position in the testing machine, and the loaded metal head contacts the inner slope of the functional cusp. The maximum breaking load before failure was recorded in Newtons (N), and mean values were calculated per group.

After the fracture resistance test, the fracture mode was assessed using a stereomicroscope (Nikon, Tokyo, Japan) at 10× magnification. The mode of failure was recorded according to a classification method by Furtado de et al. [23] and Ellakwa et al. [24]. Type I indicates minimal chipping, capable of refinishing and repair; in type II less than half the crown is lost and the replica is intact; type III indicates crown fracture through the midline with half the crown displaced or lost; and type IV shows a severe fracture of the replica and the crown.

### Statistical analysis

Data were statistically analysed using SPSS software (SPSS 20.0, SPSS, USA). Fracture resistance data were first analysed by the Kolmogorov‒Smirnov test to confirm a normal distribution, and then 2-way ANOVA was used to examine the effects of the abutment designs and the crown materials, as well as the interaction between variables. The significant differences in various groups were analysed with a 1-way ANOVA test followed by a *post hoc* Tukey’s honestly significant difference test. Fracture modes were compared using Pearson’s chi-square test. The level of significance was set at α = 0.05.

#### Scanning electron microscopy (SEM) observations

The fractured specimens were selected for examination with by SEM (NOVA NanoSEM 230, FEI, USA). The representative morphology of the crown surface of the fractured specimens was sputtered with a gold-palladium alloy by an ion sputtering equipment (Emitech K550X, Quorum, Britain) to form a conductive layer and examined by SEM with an acceleration voltage of 5 kV.

## Results

Two-way ANOVA revealed that the abutment design (F = 28.44, P = 1.52 × 10^− 8^<0.001)and the crown materials (F = 4.37, P = 0.043 < 0.05) had statistically significant effects on the fracture resistance of implant crown restoration. Table 2 shows the mean fracture loads for each group and the results of the statistical analysis. One-way ANOVA revealed that there were statistically significant differences among the six groups for the mean fracture loads (F = 15.59, P = 1.15 × 10^− 8^<0.01). The Lava Ultimate-2 mm group showed the greatest fracture resistance of 2222.74 ± 320.36 N, and the Vita amic-3 mm group showed the smallest fracture resistance of 1204.96 ± 130.50 N.

**Table 2:** Mean value and standard deviations (N) and significance of fracture load test. Different letters indicate that differences were statistically significant (p < 0.05)

	Group	Mean	Standard deviations	Significance
Vita Enamic	1 mm	1545.04	331.74	ab
	2 mm	1707.09	289.31	a
	3 mm	1204.96	130.50	b
Lava Ultimate	1 mm	1378.25	232.76	ab
	2 mm	2222.74	320.36	c
	3 mm	1383.84	208.54	ab

**Fig. 2:**
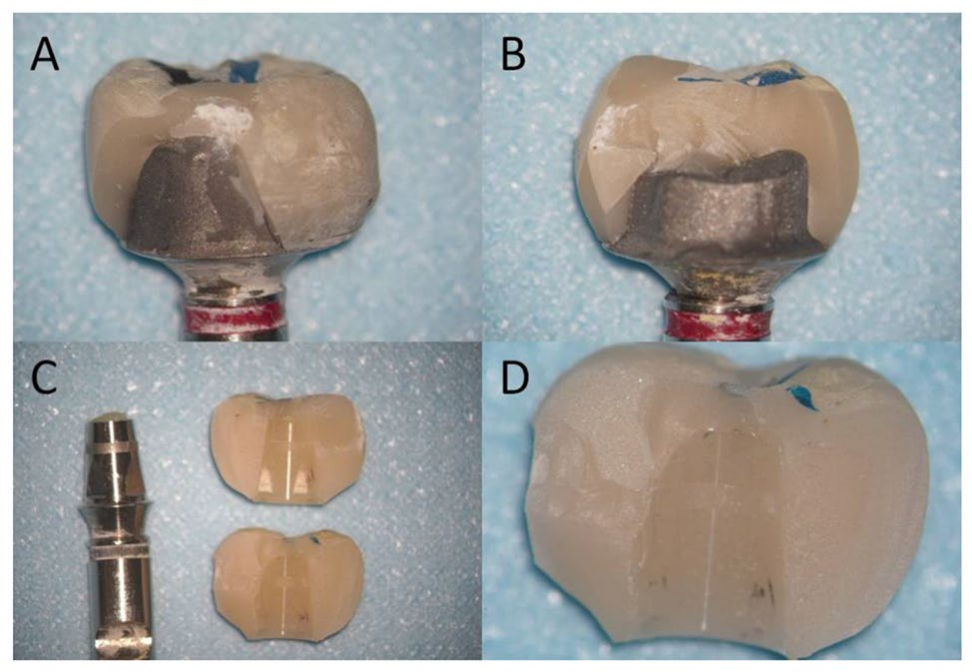
Typical fracture specimens with three abutment designs A: Vita Enamic-1 mm (Type II), B: Vita Enamic-2 mm (Type III), C and D: Vita Enamic-3 mm (Type IV). In the 1 mm (A) and 2 mm groups (B), some residual crown restorations remained on the abutments, while in the 3 mm group (C and D), the crowns completely detached from the abutments

**Fig. 3:**
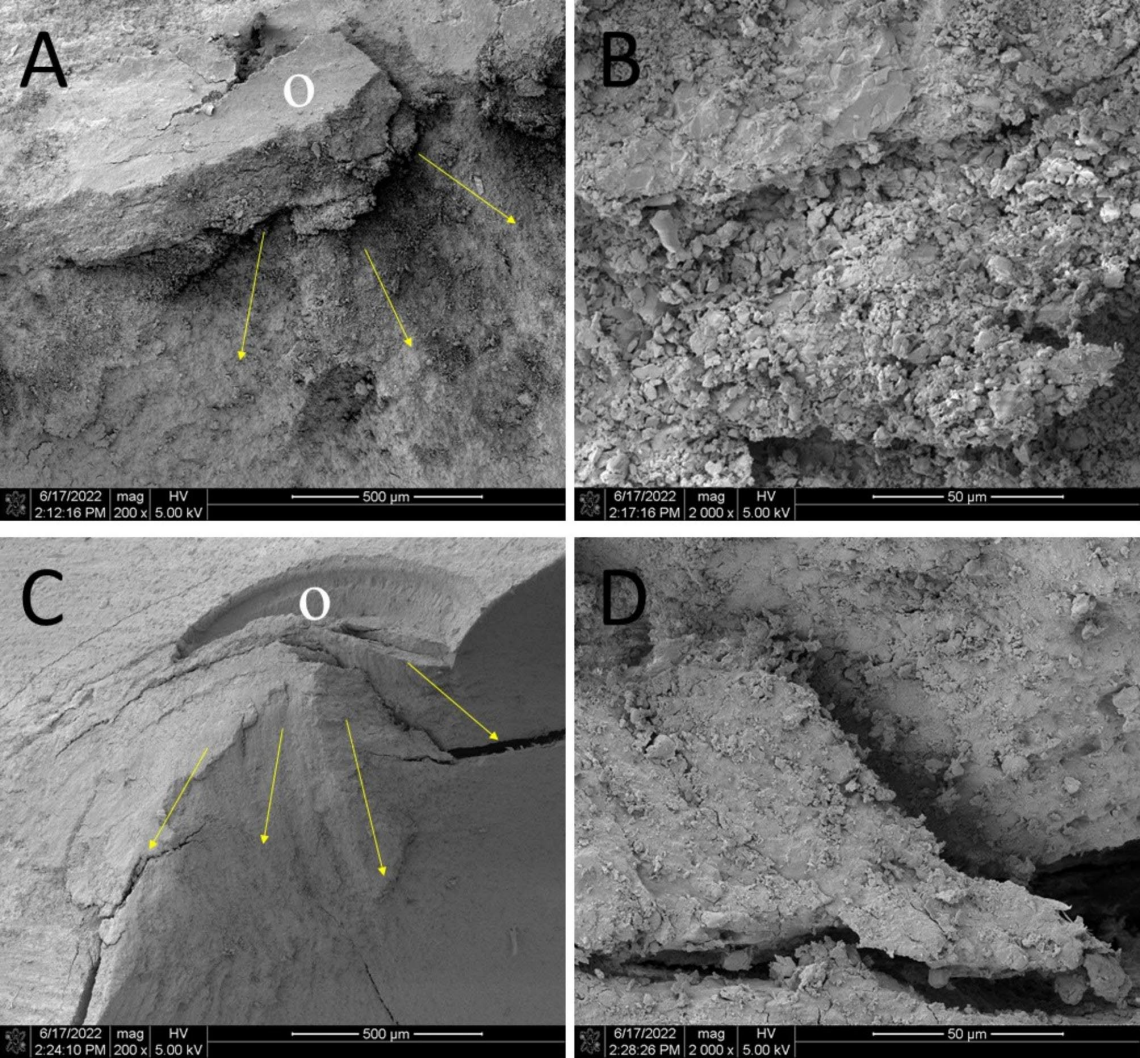
Scanning electron microscope photographs of the fractured surface A (×200) and B (× 2000): Vita Enamic-2 mm, C (×200) and D (× 2000): Lava Ultimate-2 mm. White circle o: fracture origin, arrows: direction of crack propagation. The fracture surface of Vita Enamic (A and B) showed a coarser grain structure, which was related to its double-network structure. Lava Ultimate (C and D) showed more delicate particles and a smooth fracture surface, which was closer to the fracture structure of the composite resin

The statistics of the fracture mode of the specimens are shown in Table 2. Figure 2 shows typical fracture specimens in the three abutment designs. Table 3 shows the number and pattern of failure after fracture test. Regardless of whether the crowns were made of two materials, most of the 1 and 2 mm groups underwent Type II failures, and the crown restorations were partially detached from the abutment. Most of the 3 mm groups underwent Type IV failures, and the crown restorations were completely detached from the abutment. Figure 3 shows SEM microphotographs of the fracture surfaces.

**Table 3:** Number and pattern of failure after fracture test. Different letters indicate that differences were statistically significant (p < 0.05)

		Type I	Type II	Type III	Type IV	Significance
Vita Enamic	1 mm	1	7	0	0	a
	2 mm	1	5	2	0	a
	3 mm	0	0	2	6	b
Lava Ultimate	1 mm	2	6	0	0	a
	2 mm		6	2	0	a
	3 mm	0	0	2	6	b

## Discussion

The resin-based ceramics Lava Ultimate and Vita Enamic used in this study were two resin-based ceramics with different preparation processes [25], and their mechanical properties were different. Thus, they were included in the experiment for comparison. According to the current literature, the research results on the effect of hydrofluoric acid on bonding are inconsistent. Fathy et al. [18] reported that after hydrofluoric acid treatment, the adhesion of Lava Ultimate was lower than that of air abrasion and universal adhesive group. In addition, hydrofluoric acid is a highly toxic drug with high risk, the crowns were not treated with hydrofluoric acid in this study.

Vita Enamic had a polymer-infiltrated ceramic network. The ceramic part could significantly enhance the strength of the material itself, making its performance superior to that of composite resin materials. In this study, the fracture strengths of 1 mm- and 2 mm-thick crowns were greater than that of the 3 mm-thick crowns. Rosentitt et al. [22] reported the fracture resistance of CAD/CAM-fabricated implant-supported molar crowns. They used a custom abutment similar to the one used this study, but they did not change the thickness design of the platform. In this study, the fracture resistance value of Vita Enamic was 1707.09 N, which was similar to the results (1385.5 N) of their study [22]. de Kok et al. [17] also reported Vita Enamic used on a titanium abutment was approximately 2171 N, all restorations (zirconia, resin-based and lithium disilicate materials) were in a range where it seemed that clinical application would not be restricted [17].

However, according to some studies, the strengths of resin-based ceramic materials are not sufficient to support implant crowns. Schepke et al. [26] reported that in the Lava Ultimate group, 40 restorations (80%) debonded and 3 restorations (6%) fractured within 1 year of clinical service. In all of the observed debonding cases, the residual cement was predominantly located in the Lava Ultimate crown and not on the abutment. In another article [27], the uncompromised survival rate of resin-based ceramic crowns bonded to zirconia abutments after 1 year of clinical service was only 14%. Such a low success rate may have been because the study did not use a custom rational design of the abutment, and because the study used a zirconia abutment that was not easy to bond. Therefore, resin-based ceramics cannot be simply considered for implant repair in the posterior tooth region. Zirconia abutments are often used in the aesthetic area of anterior teeth, while titanium abutments are often used in posterior teeth to withstand greater bite force. The research results of Rosentritt [22] and Preis et al. [1]. were consistent with the results of this study. They reported that most CAD/CAM materials (including resin-matrix ceramics) may be clinically applied in implant-supported crowns without restrictions. It should be noted that poor adhesion of zirconia abutments is one of the limitations.

The combination of a thick zirconia crown and a small core abutment in clinical practice rarely causes crown fracture, mainly due to the high strength of zirconia [28]. However, when using resin-matrix ceramics with low strength, it is necessary to control the shape of the abutment and the thickness of the crown; otherwise, crown fracture failure may occur [27]. This is similar to the relationship between the veneering porcelain and the metal coping of PFM [29]. A veneering porcelain with low strength should be supported by a high-strength core. The surface material is too thick and cracks too easily. If it is too thin, then it easily undergoes wear and leads to poor aesthetics. Experimental and clinical data are needed to support the relationship to balance the use of veneers and cores with different properties [30].

According to the statistical results of fracture modes, crowns with thicknesses of 1 and 2 mm mainly underwent Type I and Type II fractures. The parts detached by fractures were relatively small and could be restored by direct resin restoration. However, mainly Type III and Type IV fracture were observed for crowns with thickness of 3 mm. The crown was damaged severely, and it was impossible to repair the defects through direct application of resin in the mouth, and thus it was necessary to remove the fractured crown and replace it with a new one. Moreover, the problem of abutment deformation was present in the 3 mm thickness group but not in the 1 and 2 mm groups. This may have been related to the strength of the custom abutment. Crowns with prefabricated abutments easily lead to stress concentrations. The crowns split into two pieces from the occlusal surface, which resulted in irreparable fracture patterns. Considering this factor, designing custom abutments is recommended when using resin-matrix ceramics as implant crowns.

## Conclusions

Within the limitations of this in vitro study, it could be concluded that the fracture resistance was greater for the ceramic crown designs with 2 mm than 1 and 3 mm thick resin matrices. Most of the specimens in the 1 and 2 mm groups underwent partial crown fracture that could be repaired directly with resin. In contrast, the specimens in the 3 mm group underwent longitudinal crown fracture, and the crown detached from the abutment. The 2 mm thick resin-matrix ceramic crown and personalized abutment are an option to replace zirconia for implant crown restoration.

## Data Availability

The complete data and materials described in the research article are freely available from the corresponding author on reasonable request.

## Abbreviations

PFM: porcelain-fused-to-metal
CAD/CAM: computer-aided design/computer-aided manufacturing
SEM: scanning electron microscopy
